# Supplementary material for: Combining genomic and epidemiological data to compare the transmissibility of SARS-CoV-2 variants Alpha and Iota
Source: Commun Biol. 2022 May 11;5:439. doi: 10.1038/s42003-022-03347-3 (PMC9095641; doi:10.1038/s42003-022-03347-3)
Supplement: Supplementary file 2 — Supplementary Information [file 42003_2022_3347_MOESM2_ESM.pdf]

(a)

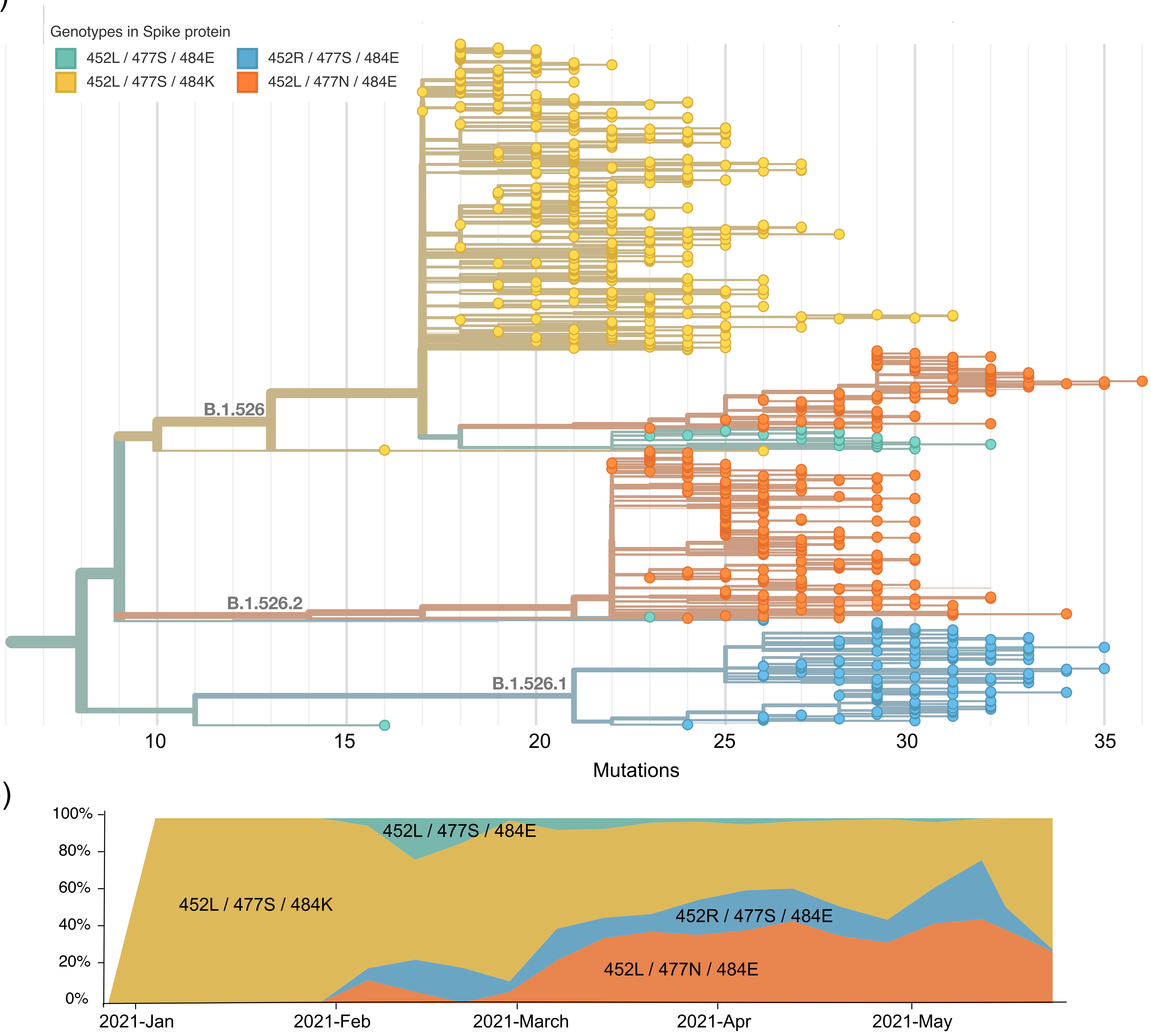

(b)

Supplementary Figure 1: Complex phylogenetic topology and synchronous epidemiology of Iota sublineages in Connecticut. (a) Maximum likelihood phylogenetic tree of Iota sublineages built using Nexstrain (nextstrain.org). (b) Normalized frequencies of Iota sublineages based on 1,011 publicly available whole genomes sampled in Connecticut.

Supplementary Table 1: Distribution of 'other' lineages in Connecticut sequenced between November 30, 2020 and May 9, 2021. Current genomic surveillance data for Connecticut are available at https://covidtrackerct.com. Variants of Concern are shown in red and Variants of Interest are shown in blue (based on CDC classifications as of June 10, 2021).

Lineage	Count		Lineage	Count
A	1		B.1.36.10	1
A.2.5	1		B.1.361	1
A.23.1	1		B.1.369	1
B	1		B.1.37	1
B.1	25		B.1.375	2
B.1.1	12		B.1.400	2
B.1.1.1	3		B.1.409	1
B.1.1.192	7		B.1.420	1
B.1.1.28	3		**B.1.427**	**21**
B.1.1.348	2		**B.1.429**	**42**
B.1.1.372	1		B.1.509	3
B.1.1.416	1		B.1.517	106
B.1.1.420	1		B.1.517.1	1
B.1.1.434	16		B.1.523	2
B.1.1.486	19		**B.1.525**	**4**
B.1.1.519	17		B.1.543	1
B.1.110.3	4		B.1.551	1
B.1.111	10		B.1.568	6
B.1.177	1		B.1.575	55
B.1.2	159		B.1.575.1	1
B.1.234	2		B.1.577	2
B.1.240	2		B.1.588	1
B.1.243	46		B.1.596	9
B.1.265	1		B.1.604	1
B.1.280	1		**B.1.617.2**	**2**
B.1.298	1		B.1.621	1
B.1.311	2		C.37	5
B.1.314	1		**P.1**	**40**
B.1.320	1		R.1	33
B.1.349	4		R.2	3
**B.1.351**	**17**			

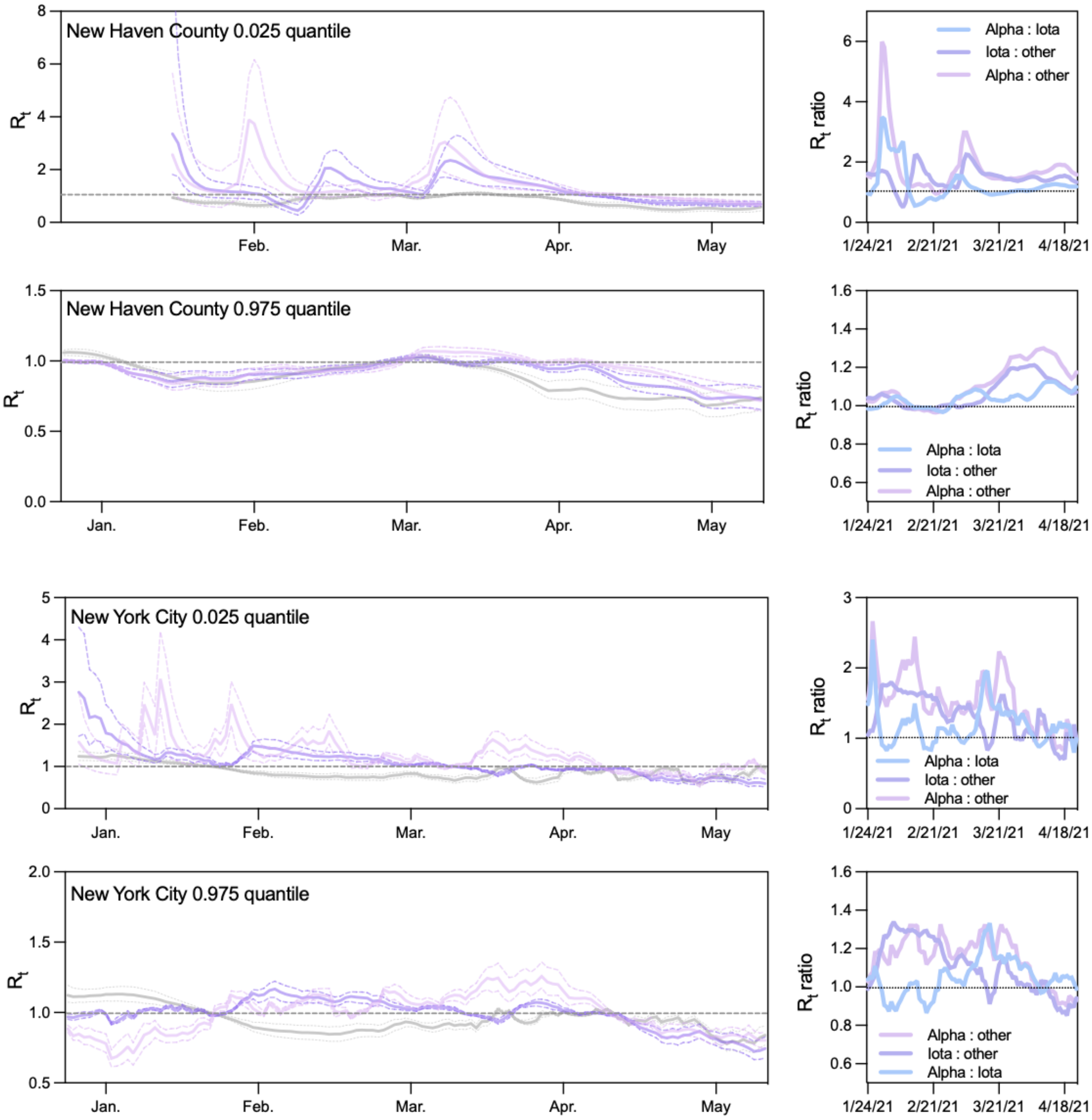

Supplementary Figure 2: Effective reproduction number (R_t) estimates for circulating variants using lower and upper Jeffreys intervals (0.025 and 0.975 quantiles, respectively).

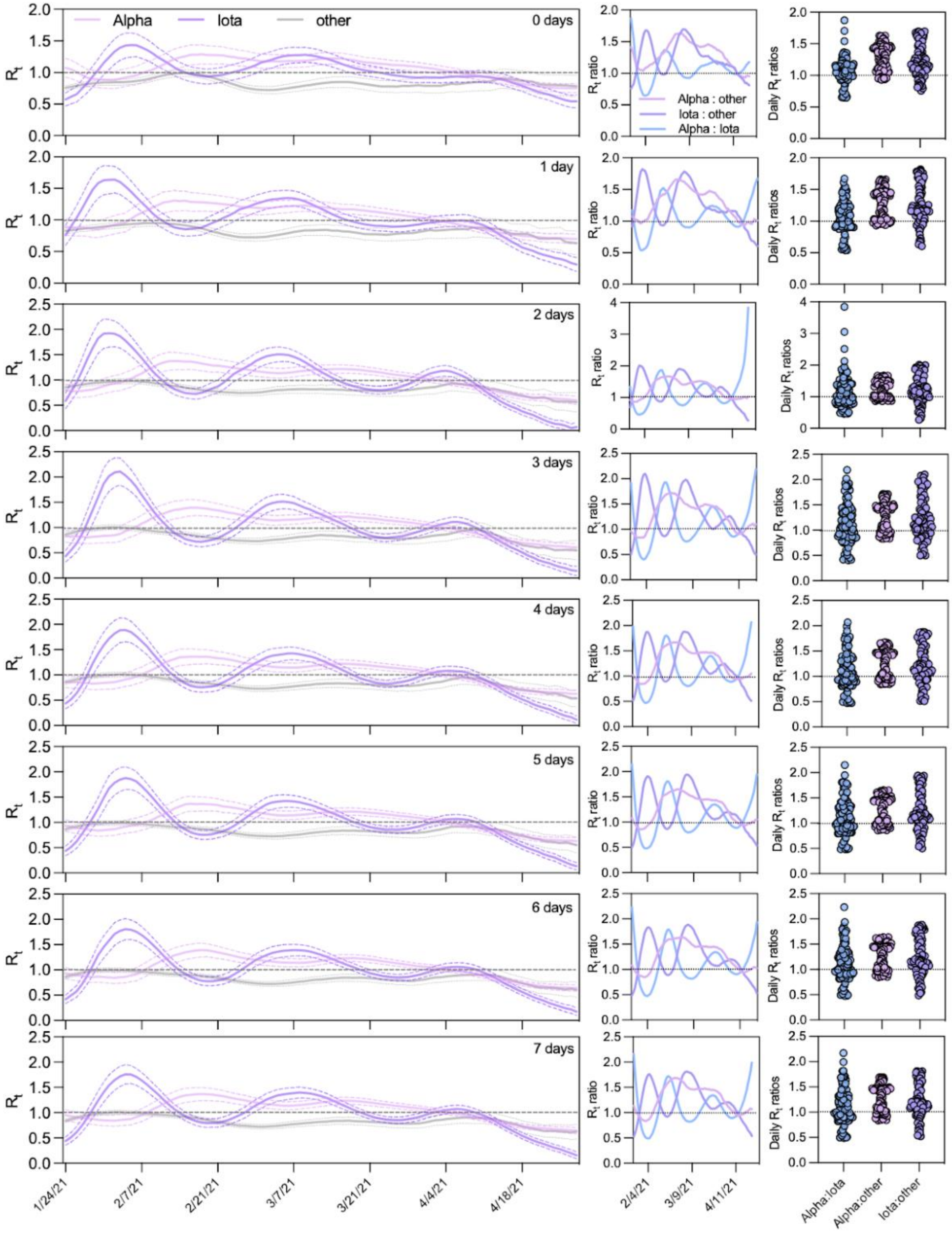

Supplementary Figure 3: R_t estimates for Alpha, Iota, and other variants in New Haven County. R_t estimates were calculated using the R package 'EpiNow2' assuming 0-7 days of reporting delays as indicated by the number in the upper right corner of each graph. Solid lines indicate the median daily estimate, while dotted lines indicate the standard deviation.

Supplementary Table 2: Size of replicate datasets

	Alpha			Iota		
rep.	Downsampled (functional duplicates removed)	Outliers removed	Scaled by 0.1x	Downsampled (functional duplicates removed)	Outliers removed	Scaled by 0.1x
1	17,897	17,877	2,970	12,836	12,830	1,726
2	17,939	17,916	2,975	12,840	12,829	1,706
3	17,959	17,937	3,194	12,837	12,825	1,717
4	17,922	17,902	2,970	12,838	12,829	1,704
5	17,927	17,907	2,979	12,835	12,824	1,713

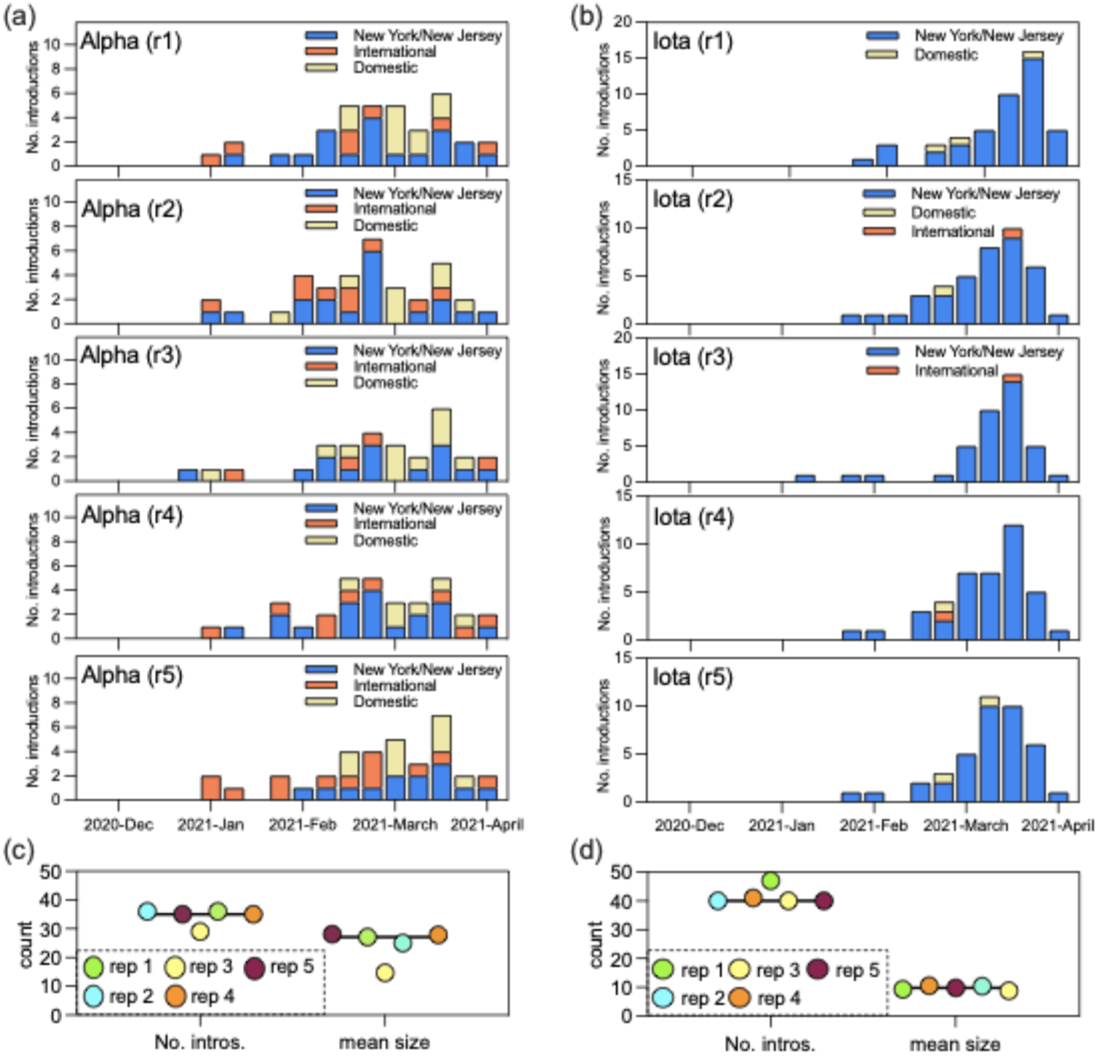

Supplementary Figure 4: Discrete phylogeographic results for downsampled replicates. (**a, b**) Number of introductions by week for Alpha (a) and Iota. (**c,d**) Summary of number and mean size of introductions for Alpha (c) and Iota (d) replicates.
