# Supplementary material for: Combining genomic and epidemiological data to compare the transmissibility of SARS-CoV-2 variants Alpha and Iota
Source: Commun Biol. 2022 May 11;5:439. doi: 10.1038/s42003-022-03347-3 (PMC9095641; doi:10.1038/s42003-022-03347-3)
Supplement: Supplementary file 3 — Reporting Summary [file 42003_2022_3347_MOESM3_ESM.pdf]

nature portfolio

Corresponding author(s): Mary Petrone, Nathan Grubaugh

Last updated by author(s): Mar 3, 2022

Reporting Summary

Nature Portfolio wishes to improve the reproducibility of the work that we publish. This form provides structure for consistency and transparency in reporting. For further information on Nature Portfolio policies, see our Editorial Policies and the Editorial Policy Checklist.

Statistics

For all statistical analyses, confirm that the following items are present in the figure legend, table legend, main text, or Methods section.

n/a	Confirmed	
☐	☒	The exact sample size (*n*) for each experimental group/condition, given as a discrete number and unit of measurement
☐	☒	A statement on whether measurements were taken from distinct samples or whether the same sample was measured repeatedly
☐	☒	The statistical test(s) used AND whether they are one- or two-sided<br>*Only common tests should be described solely by name; describe more complex techniques in the Methods section.*
☐	☒	A description of all covariates tested
☐	☒	A description of any assumptions or corrections, such as tests of normality and adjustment for multiple comparisons
☐	☒	A full description of the statistical parameters including central tendency (e.g. means) or other basic estimates (e.g. regression coefficient) AND variation (e.g. standard deviation) or associated estimates of uncertainty (e.g. confidence intervals)
☐	☒	For null hypothesis testing, the test statistic (e.g. *F*, *t*, *r*) with confidence intervals, effect sizes, degrees of freedom and *P* value noted<br>*Give P values as exact values whenever suitable.*
☐	☒	For Bayesian analysis, information on the choice of priors and Markov chain Monte Carlo settings
☒	☐	For hierarchical and complex designs, identification of the appropriate level for tests and full reporting of outcomes
☒	☐	Estimates of effect sizes (e.g. Cohen's *d*, Pearson's *r*), indicating how they were calculated

Our web collection on statistics for biologists contains articles on many of the points above.

Software and code

Policy information about availability of computer code

Data collection	We used pangolin v.2.4.2 to assign lineages to the SARS-CoV-2 genomes included in our variant frequency estimates.
Data analysis	The Jeffreys intervals for variant frequencies were calculated using the package "DescTools" in R v4.0.1. All of the Rt estimates were calculated using the "EpiEstim" package in Rv4.0.1, and we used a smoothing spline to smooth the daily Rt curves with the package "stat" in R v4.0.1. We aligned the genomes using MAFFT and constructed maximum likelihood trees using IQTree. Time-resolved trees were inferred using TreeTime v.0.8.0, and we performed our Bayesian phylogeographic analysis using BEAST v.1.10.5. We tabulated the number of introductions into Connecticut using the "exploded tree" script implemented with baltic v0.1.6.

For manuscripts utilizing custom algorithms or software that are central to the research but not yet described in published literature, software must be made available to editors and reviewers. We strongly encourage code deposition in a community repository (e.g. GitHub). See the Nature Portfolio guidelines for submitting code & software for further information.

Data

Policy information about availability of data

All manuscripts must include a data availability statement. This statement should provide the following information, where applicable:

- Accession codes, unique identifiers, or web links for publicly available datasets
- A description of any restrictions on data availability
- For clinical datasets or third party data, please ensure that the statement adheres to our policy

All of the genomic data used for the analyses in this manuscript are available on GISAID (gisaid.org). We gratefully acknowledge all of the laboratories that obtained the clinical specimen and generated the SARSCoV-2 genomes used in our analyses. A complete list of genome accessions and contributing laboratories is included in

the supplement. All files associated with our Rt estimates and phylogenetic analysis may be found in our Github repository (https://github.com/grubaughlab/paper_2021_B117vsB1526).

Field-specific reporting

Please select the one below that is the best fit for your research. If you are not sure, read the appropriate sections before making your selection.

☒ Life sciences ☐ Behavioural & social sciences ☐ Ecological, evolutionary & environmental sciences

For a reference copy of the document with all sections, see nature.com/documents/nr-reporting-summary-flat.pdf

Life sciences study design

All studies must disclose on these points even when the disclosure is negative.

Sample size	To track the frequency of SARS-CoV-2 lineages over time, we used available sequencing data from three locations: New Haven County (n = 2,086), Fairfield County (n = 612), and New York City (n = 4,528). For our phylogenetic analysis, we incorporated 2,970 B.1.1.7 genomes and 1,704 B.1.526 genomes. We selected genomes from the public repository GISAID based on the reported number of COVID-19 deaths per country or US state.
Data exclusions	For our lineage frequency estimates, we excluded genomes that were targeted for sequencing due to S-gene target failure or any other related reason.
Replication	We replicated our variant-specific phylogeographic analysis five times per lineage. We found that the exact number of inferred introductions varied slightly across replications, but the average volume and source of introductions into Connecticut did not qualitatively differ across replicates.
Randomization	For our phylogeographic analysis, we randomly selected whole SARS-CoV-2 genomes available on GISAID using a pseudo-random number generator implemented in Python, normalizing the volume of genomes per country or US state by the number of COVID-19 deaths reported in each region.
Blinding	Blinding was not relevant to this study because we used de-identified clinical samples.

Reporting for specific materials, systems and methods

We require information from authors about some types of materials, experimental systems and methods used in many studies. Here, indicate whether each material, system or method listed is relevant to your study. If you are not sure if a list item applies to your research, read the appropriate section before selecting a response.

Materials & experimental systems

n/a	Involved in the study	
☒	☐	Antibodies
☒	☐	Eukaryotic cell lines
☒	☐	Palaeontology and archaeology
☒	☐	Animals and other organisms
☐	☒	Human research participants
☒	☐	Clinical data
☒	☐	Dual use research of concern

Methods

n/a	Involved in the study	
☒	☐	ChIP-seq
☒	☐	Flow cytometry
☒	☐	MRI-based neuroimaging

Human research participants

Policy information about studies involving human research participants

Population characteristics	Yale University<br>We received clinical samples from confirmed SARS-CoV-2 positive individuals from routine testing provided by Yale New Haven Hospital, Yale Pathology Laboratory, "Yale Campus Study", Connecticut Department of Public Health, and Murphy Medical Associates. Jackson Laboratory<br>Clinical samples were received in The Jackson Laboratory Clinical Genomics Laboratory (CGL) as part of a statewide COVID-19 surveillance program, with the majority of samples representing asymptomatic screening of nursing home and assisted living facility residents and staff. New York State Department of Health, Wadsworth Center Respiratory swabs positive for SARS-CoV-2 were sent to the Wadsworth Center from collaborating clinical laboratories across New York State as part of an enhanced genomic surveillance program initiated by the New York State Department of Health in December 2020.<br><br>In all cases, clinical samples were de-identified so that individual demographic information was not disclosed to the sequencing laboratories.

Recruitment	We did not recruit participants because all clinical samples were obtained as remnants from diagnostic testing.
Ethics oversight	Yale University<br>The Institutional Review Board from the Yale University Human Research Protection Program determined that the RT-qPCR testing and sequencing of de-identified remnant COVID-19 clinical samples obtained from clinical partners conducted in this study is not research involving human subjects (IRB Protocol ID: 2000028599).<br><br>Jackson Laboratory<br>The Institutional Review Board of The Jackson Laboratory determined that use of de-identified residual COVID-19 clinical samples obtained from the Clinical Genomics Laboratory for RT-qPCR testing and sequencing for this study is not research involving human subjects (IRB Determination: 2020-NHSR021).<br><br>New York State Department of Health, Wadsworth Center<br>Residual portions of respiratory specimens from individuals who tested positive for SARS-CoV-2 by RT-PCR were obtained from the Wadsworth Center and partnering clinical laboratories. This work was approved by the New York State Department of Health Institutional Review Board, under study numbers 02-054 and 07-022.

Note that full information on the approval of the study protocol must also be provided in the manuscript.
